# Photodetectors Based on Organic–Inorganic Hybrid Lead Halide Perovskites

**DOI:** 10.1002/advs.201700256

**Published:** 2017-09-15

**Authors:** Jiachen Zhou, Jia Huang

**Affiliations:** ^1^ School of Materials Science and Engineering Tongji University Shanghai 201804 P. R. China

**Keywords:** flexible electronics, organic–inorganic hybrid lead halide perovskites, photoconductors, photodiodes, phototransistors

## Abstract

Recent years have witnessed skyrocketing research achievements in organic–inorganic hybrid lead halide perovskites (OIHPs) in the photovoltaic field. In addition to photovoltaics, more and more studies have focused on OIHPs‐based photodetectors in the past two years, due to the remarkable optoelectronic properties of OIHPs. This article summarizes the latest progress in this research field. To begin with, the factors influencing the performance of photodetectors are discussed, including both internal and external factors. In particular, the channel width and the incident power intensities should be taken into account to precisely and objectively evaluate and compare the output performance of different photodetectors. Next, photodetectors fabricated on single‐component perovskites in terms of different micromorphologies are discussed, namely, 3D thin‐film and single crystalline, 2D nanoplates, 1D nanowires, and 0D nanocrystals, respectively. Then, bilayer structured perovskite‐based photodetectors incorporating inorganic and organic semiconductors are discussed to improve the optoelectronic performance of their pristine counterparts. Additionally, flexible OIHPs‐based photodetectors are highlighted. Finally, a brief conclusion and outlook is given on the progress and challenges in the field of perovskites‐based photodetectors.

## 1 Introduction

Organic–inorganic hybrid lead halide perovskites (OIHPs) have recently emerged as promising materials for the optoelectronic community. In this sense, stunning achievements have been made in photovoltaic field in terms of perovskite solar cells (PSCs) with certified power conversion efficiency (PCE) exceeding 22%.^1^, ^2^, ^3^, ^4^, ^5^, ^6^, ^7^, ^8^, ^9^, ^10^, ^11^ Over the past few years, OIHPs have been attracting the attention of a great number of scientific researchers with the aim to study the fundamental photophysics and practical applications of these materials. As a result, a variety of unprecedented fruits have been yielded, which has been described as a “perovskite fever.”^12^, ^13^, ^14^, ^15^, ^16^ More importantly, the skyrocketing PCE of PSCs does not seem to hit an insurmountable bottleneck, further revealing the significance of OIHPs‐related studies.^17^, ^18^, ^19^ In addition to intensive studies on PSCs, OIHPs also hold great potential in many other fields such as field effect transistors (FETs), light emitting diodes (LEDs), and photodetectors owing to the remarkable optoelectronic characteristics of OIHPs (e.g., high external quantum efficiency (EQE) over a wide absorption spectrum, high absorption coefficients, tunable optical band gaps, low trap density, reduced charge carrier recombination rates, and long charge carrier diffusion lengths and lifetimes).^20^, ^21^, ^22^, ^23^, ^24^, ^25^, ^26^ Among these photovoltaic or optoelectronic devices, photodetectors (PDs), which convert light into electric signals, have been widely employed as fundamental devices in the optical communication, automatic control, video imaging, biochemical sensors, night vision, missile guidance, and many other industrial or military fields.^27^, ^28^, ^29^ It is the significance of photodetectors and the remarkable optoelectronic characteristics of OIHPs that more and more studies have been reported on OIHPs‐based PDs, especially in the past two years.

In this review, we first examine the factors influencing the performance of PDs including internal factors (i.e., chemical compositions, crystalline structures, material micromorphologies, device architectures, and perovskite/electrode interface) and external factors (i.e., testing conditions and environment). Second, we describe the synthesis process, device fabrication, sensing mechanisms, and optoelectronic performance of PDs in terms of their different micromorphologies, namely, 3D thin‐film and single crystalline, 2D nanoplates, 1D nanowires (NWs), and 0D nanocrystals, respectively. Third, we discuss bilayer‐structured OIHPs‐based PDs incorporating inorganic and organic semiconductors, with the aim of further revealing the sensing mechanisms and improving the electrical behavior of these devices. Fourth, flexible OIHPs‐based PDs are summarized with a focus on transparent, lightweight, and environmentally friendly devices with excellent mechanical integrity and remarkable electrical endurance properties. Finally, we provide a brief conclusion and outlook on both the achievements and challenges of OIHPs‐based PDs. We hope this paper can provide readers with a comprehensive perspective on OIHPs‐based PDs, stimulating new thinking and exploration as well as promoting the development of optoelectronic fields.^14^, ^30^

## 2 Factors Influencing Photodetectors Performances

To quantitatively evaluate the output performance of PDs, several figures of merit will be introduced to make a clear and accurate comparison of the optoelectronic properties of the reported PDs.^31^, ^32^

(1) The EQE indicates the photon–electron conversion efficiency and is calculated as follows

(1) $$\mathrm{EQE}\;\;=\;\;\frac{Rh\upsilon }{e}$$

where *hυ* is the energy of incident photon and e is elementary electron charge.^31^

(2) The photoresponsivity (*R*) is closely related to the quantum yield of PDs and is defined as follows

(2) $$R\;\;=\;\;\frac{I_{\mathrm{light}}\;\;-\;I_{\mathrm{dark}}}{P_{\mathrm{in}}A}$$

where *I*_light_, *I*_dark_, *P*_in_, and *A* are photocurrent, dark current, incident light intensity, and active area, respectively.^31^, ^32^

(3) The detectivity (D*) is defined as follows

(3) $$D*\;\;=\;\;\frac{\sqrt{A\Delta f}}{\mathrm{NEP}}$$

where Δ*f* is the detection bandwidth and NEP is the noise equivalent power. When the shot noise from *I*_dark_ is the main component of the overall PD noise, this expression can be simplified as below^32^, ^33^, ^34^

(4) $$D*\;\;\approx \;\;\frac{\sqrt{2}R}{\sqrt{2eI_{\mathrm{dark}}}}$$

(4) The *I*_light_/*I*_dark_ ratio (or on/off ratio) is calculated as the *I*_light_ to *I*_dark_ ratio at fixed incident power, wavelength, and bias voltage conditions, which reflects the photosensitivity of a device.
(5) The temporal response time provides the response speed of a device toward an incident light and is expressed as the rise (τ_rise_) and decay (τ_decay_) times. This parameter is closely related to complicated charge trapping/detrapping and recombination processes.^31^
(6) The photoconductive gain (*G*) indicates the number of charge carriers travelling through an external circuit per incident photon and can be determined as follows
  (5) $$G\;\;=\;\;\frac{\tau_{l}}{\tau_{t}}\;\;=\;\;\frac{\tau_{l}}{d^{2}/\mu V}$$

where τ_l_ is the charge lifetime of holes, τ_t_ is the charge carrier transit time of electrons, *d* is the device thickness, μ is the charge carrier mobility, and *V* is the bias voltage, respectively.^35^

(7) The linear dynamic range (LDR) or photosensitivity linearity provides a linear relationship between the *I*_light_ and *P*_in_ in a certain range, and is always expressed in logarithmic scale as follows
  (6) $$\mathrm{LDR}\;\;=\;\;20\;\log\;\frac{J_{\mathrm{ph}}^{*}}{J_{d}}$$

where $J_{\mathrm{ph}}^{*}$ is the photocurrent density measured at a light illumination of 1 mW cm^−2^ and *J*_d_ is the dark current density.^31^

When the above figures of merit present outstanding values, the PDs show good output performance, and this is determined by PDs themselves. Thus, in this section, we will discuss in detail the factors affecting the optoelectronic performance of PDs, including both internal factors (i.e., chemical composition, crystalline structure, material micromorphology and device architecture, and perovskite/electrode interface) and external factors (i.e., testing conditions and water‐vapor and oxygen invasive environment). In turn, a good understanding of these factors is conducive to designing and fabricating desired PDs to realize excellent output performances.

### 2.1 Internal Factors

#### 2.1.1 Chemical Compositions and Crystalline Structures

**Figure 1:**
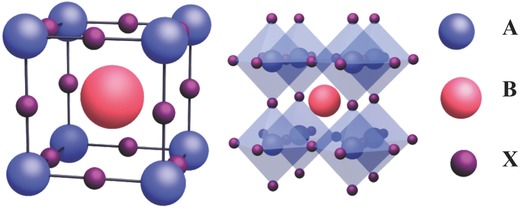
Unit cell of an ideal cubic perovskite ABX_3_ (left), and their extended crystalline structured connected by corner‐sharing [BX_6_]^4−^ octahedra (right). Reproduced with permission.^39^

The terminology “perovskite” used here does not refer to the calcium titanate (CaTiO_3_) mineral but to a class of semiconducting materials with CaTiO_3_‐like crystalline structure and has a generalized chemical formula of ABX_3_, where A can be an inorganic ion (e.g., Cs^+^ and Rb^+^) or an aliphatic/aromatic moiety (e.g., CH_3_NH_3_^+^, HC(NH_2_)_2_^+^), B denotes a bivalent transition metal ion (e.g., Pb^2+^, Sn^2+^, Ge^2+^, Mn^2+^, Cu^2+^) and X represents a halide ion (e.g., Cl^−^, Br^−^, and I^−^).^36^, ^37^ In a typical unit cell of ABX_3_ (see **Figure** **1**), the A cations occupy eight corners, the B ions stand at the body center and the X anions locate at six face center. The B ion usually coordinates with six X anions to form [BX_6_]^4−^ octahedron, which are connected each other by shared corners and extended to 3D perovskites.^22^, ^37^ In order to estimate the formability and stability of potential perovskites, Goldschmidt tolerance factor (*t*) is considered and described as follows

(7) $$t\;\;=\;\;\frac{r_{A}+\;r_{X}}{\sqrt{2}\left(r_{B}\;\;+r_{X}\right)}$$

where *r*_A_, *r*_B_, and *r*_X_ are the ionic radii of A, B, and X, respectively.^36^, ^37^ This semiempirical formula indicates that *t* is in the range of 0.85 < *t* < 1.11 for *Pm*3*m* cubic perovskites and deviations from this range can lead to distorted structures, changeable crystalline symmetries, or reduced dimensionalities.^20^, ^38^, ^39^, ^40^

The octahedral factor has also been proposed as a guideline to predict the formability of perovskites. It is defined as the ratio of *r*_B_ to *r*_X_. When *r*_B_/*r*_X_ is less than 0.442, a perovskite structure is hard to exist with unstable [BX_6_]^4−^ octahedra. By combining these two formability criteria, nearly all the perovskites with varying chemical compositions can be precisely predicted, designed, and synthesized. For example, tetragonal methylammonium lead iodide (CH_3_NH_3_PbI_3_, MAPbI_3_) with $r_{{\mathrm{MA}}^{+}}$ = 0.18 nm, $r_{{\mathrm{Pb}}^{2+}}$ = 0.12 nm, and $r_{I^{-}}$ = 0.22 nm is successfully formed at *t* and *r*_B_/*r*_X_ values of ≈ 0.83 and 0.55, respectively. A clear and thorough understanding of the electronic states of the materials is a prerequisite to reveal the physical mechanisms behind the device performance. Therefore, the intriguing electronic properties of perovskite materials have been deeply investigated based on their unique crystalline structure. Previous theoretical calculations and computational analysis have revealed that the energy levels of MAPbI_3_ compromise highest occupied molecular orbital derived from σ‐antibonding state of Pb 6s‐I 5p orbitals and lowest unoccupied molecular orbital originated from σ‐antibonding and π‐antibonding state of Pb 6p‐I 5s and Pb 6p‐I 5p orbitals, respectively. Thus, the electronic properties of MAPbI_3_ mainly arise from the inorganic [BX_6_]^4−^ framework, with A contributing to a low extent.^36^, ^37^, ^40^ Thus, the optical band gap can be tuned by varying B and X, and this can be explained associated change in the B‐p and X‐p orbitals, which results in chemical versatility and expected multifunctionality.^14^, ^36^, ^41^

**Figure 2:**
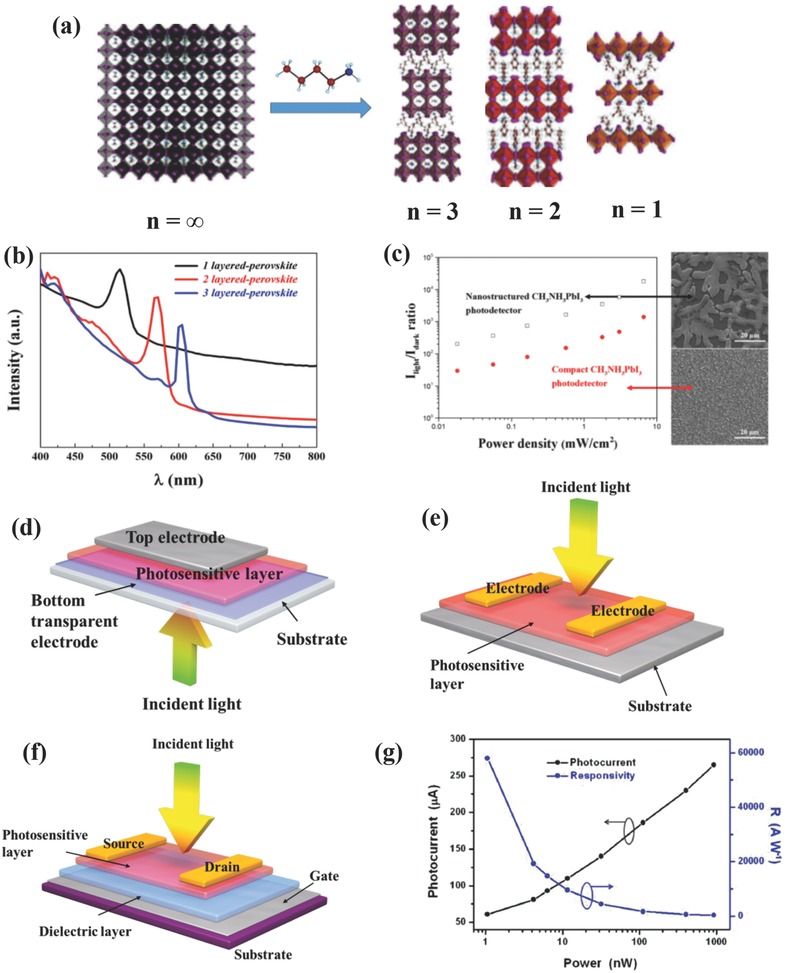
a) Crystalline structures of layer‐structured OIHPs analogues (BA)_2_(MA)*_n_*_−1_Pb*_n_*I_3_ *_n_*_+1_ (*n* = 1, 2, and 3) derived from 3D perovskites. Reproduced with permission.^51^ Copyright 2016, American Chemical Society (ACS); b) UV–vis absorption spectrum of (BA)_2_(MA)*_n_*_−1_Pb*_n_*I_3_ *_n_*_+1_ (*n* = 1, 2, and 3). Reproduced with permission.^54^ Copyright 2016, ACS; c) Comparison of the *I*_light_/*I*_dark_ ratio at different light illumination and the film morphology between the island‐structured and the compact perovskite thin film. Reproduced with permission.^68^ Copyright 2015, ACS. Schematics of the device structures of: d) photodiode with vertical configuration, e) photoconductor, and f) phototransistor (bottom‐gate/top‐contact). g) Photocurrent and photoresponsivity versus different incident powers at a fixed wavelength. Reproduced with permission.^85^ Copyright 2015, Wiley‐VCH.

From the above analysis, it can be safely concluded that chemically versatile multidimensional perovskite structures can be obtained by modifying the element or component combinations as long as the ABX_3_ perovskite structure is maintained and the charge neutrality is satisfied. For the most commonly substituted X position, a gradual variation from Cl^−^ to I^−^ resulted in: (i) crystalline structures shifting from cubic to tetragonal at room temperature; (ii) red‐shifted absorption peaks; and (iii) reduced optical band gaps, which can be ascribed to the competition between the ionic and covalent characters of the M–X bonds.^37^ This chemical tunability of perovskites enabled wide spectral absorption characteristics ranging from ultraviolet (UV) to near infrared (NIR), with the as‐synthesized OIHPs presenting vivid colors of and different potential optoelectronic applications.^42^, ^43^, ^44^ In the case of the B element, tin (Sn), germanium (Ge), bismuth (Bi), copper (Cu), and other elements have been chosen in replacement of Pb to avoid toxicity to living organisms and environmental issues. Sn‐ and Ge‐based OIHPs demonstrated promising potential for lead free perovskites devices since they belong to the same IV A group of Pb. Unfortunately, Sn^2+^ (Ge^2+^) suffers from fatal oxidation to Sn^4+^ (Ge^4+^), thereby greatly limiting the device performance and its practical applications.^45^, ^46^, ^47^ Cu‐based OIHPs such as (MA)_2_CuCl_4_ presents a unique 2D layer structure derived from a 3D structure as a result of the smaller ionic radius of Cu^2+^.^48^ Despite Cu^2+^ is stable in air, the performances of the resultant devices is still far from satisfactory because of its relative large optical band gap and poor interlayer charge transport.^17^ With regard to A cation, although it contributes little to the overall electronic properties of perovskites, its size may cause distortion of the crystalline structure, thereby affecting the electronic properties of the perovskites. In this sense, inorganic cesium ($r_{{\mathrm{Cs}}^{+}}$ = 0.17 nm), methylammonium ($r_{{\mathrm{MA}}^{+}}$ = 0.18 nm), and formamidinium ($r_{{\mathrm{FA}}^{+}}$ = 0.20 nm) ions are widely used as A cations. These materials showed larger 3D symmetry and lower optical band gaps for greater light harvest while increasing the size of the A cation. However, excessively bulky ions will lead to the collapse of 3D symmetry, and the 3D framework will transform into reduced 2D, 1D, or 0D dimensionalities.^49^ For example, large A cations were incorporated in perovskite structures to form 2D slabs by “slicing” original the 3D crystalline structure along specific crystallographic orientations.^37^ The Ruddlesden–Popper type perovskite, which has a chemical formula of (RNH_3_)_2_(CH_3_NH_3_)*_n_*_−1_M*_n_*X_3_ *_n_*_+1_ (R is an alkyl or aromatic moiety with at least four carbon atoms, M is a transition metal ion, and X is halide ion) is a 2D layer‐structured perovskite material in which the inorganic [MX_6_]^4−^ octahedra are sandwiched by bilayers of RNH_3_^+^ via weak intermolecular forces.^50^, ^51^ As shown in **Figure** **2**a, when *n* equals to 1, the structure turns out to be an ideal “quantum well” with the thinnest inorganic slabs being intercalated in organic chains. This structure approaches the 3D parent perovskite when *n* = ∞.^52^, ^53^ The 2D analogues exhibited red‐shifted absorption peaks (Figure 2b) toward that of 3D counterpart with the increase of inorganic layer (*n*), and their corresponding PDs have realized selective detection of different wavelengths with fast response times (millisecond scale).^54^ Moreover, these layer‐structured OIHPs displayed better resistance to moisture than their 3D counterpart in practical applications mainly because the long organic hydrophobic chains protect inorganic [MX_6_]^4−^ from water vapor.^50^, ^55^

#### 2.1.2 Material Micromorphologies

Apart from the chemical composition and crystalline structure, the material micromorphology plays an important role in determining the ultimate optoelectronic performance of PDs. Special micromorphologies such as 2D nanoplates, 1D NWs, 0D nanocrystals, nanonets, and inverse opals‐type structures have been carefully studied and showed superior optical properties and excellent electrical characteristics.^52^, ^53^, ^56^, ^57^, ^58^, ^59^, ^60^, ^61^, ^62^, ^63^, ^64^, ^65^ These structures will be discussed in detail in the following sections. OIHPs with inverse opals micromorphology have demonstrated various colors with tunable photonic stop bands within the visible spectrum by using polystyrene (PS) templates of different diameters. These novel light‐absorbing materials hold great promise for PSCs and other optoelectronic fields given advantages owing to their large surface area, short diffusion length, and fast transport channel characteristics.^63^, ^65^, ^66^, ^67^ Micrometer‐sized and island‐like OIHPs have been successfully prepared by controlling the content of MAPbCl_3_ in the precursor solution, and the corresponding PDs showed an ultrahigh *I*_light_/*I*_dark_ ratio of 10^4^ as compared to a compact thin film device (10^3^) and a fast response time (< 50 ms). These excellent properties derived from smooth island‐structured networks (Figure 2c) with fewer grain boundaries and lower trap densities. Moreover, these devices were thermally stable up to 100 °C without any obvious photocurrent decay.^68^

#### 2.1.3 Device Architectures

PDs can be categorized into photodiodes, photoconductors, and phototransistors based on their different device architectures. In the case of photodiodes (see Figure 2d), a photovoltaic‐like vertical configuration is favorably adopted involving the photovoltaic effect (i.e., photogenerated electrons and holes within a photosensitive layer are separated and transported toward opposite electrodes via a built‐in electric field).^32^ This kind of devices usually exhibit low driving voltage, low dark current, fast response speed, and enhanced separation efficiency of electron–hole pairs at low reverse bias under illumination. In the case of photoconductors, as shown in Figure 2e, the photosensitive layer serves as a channel between the two lateral metal electrodes, enabling a relatively simple sensing mechanism for the photon‐electron conversion process denoted as photoconductive effect (i.e., photogenerated charge carriers in the channels are separated and collected by electrodes at a bias voltage). This device architecture is easy to fabricate. In addition, transparent electrodes are not indispensable for this type of devices, thereby representing a great advantage in terms of the range of materials selection, especially for flexible optoelectronics. However, these planar‐structured devices usually suffer from slow photoresponse, low photosensitivity, and noticeable electrical hysteresis, which can be overcome via photogating effect and other methods.^69^ In the case of phototransistors (Figure 2f), a gate electrode and a dielectric layer are added to the devices with the aim to reduce the noise current, amplify the electrical signals, and improve the corresponding figures of merit like *R* and *G* values. By further incorporating OIHPs with other high‐mobility semiconductors, a photogating effect is induced such that one of the charge carriers (i.e., electrons or holes) generated in the perovskites are transferred to high‐mobility semiconductors functioning as a charge transport channel, while another charge carriers trapped in the local states serve as a gate to modulate the conductance of the perovskite via capacitive coupling.^70^, ^71^ These different device architectures will lead to significant differences in terms of optoelectronic performance. By taking *R* as an example, it *R* can be constant for photodiodes in a certain range of light intensity, which is denoted by

(8) $$R\;\;=\;\;\frac{\Delta I}{P_{\mathrm{in}}A}\;\;\propto \;\;\frac{WL}{WL}\;\;\equiv \;\;\mathrm{Const}.$$

For photoconductors and phototransistors, however, *R* is expressed as

(9) $$R\;\;=\;\;\frac{\Delta I}{P_{\mathrm{in}}A}\;\;\propto \;\;\frac{\frac{W}{L}}{WL}\;\;\propto \;\;\frac{1}{L^{2}}$$

where *W* is channel length and *L* is channel width.

It is evident that *R* values in photodiodes can be constant, and nearly independent from the size and dimensions of device. However, in case of photoconductors and phototransistors with horizontal electrode alignment, *R* can be potentially enhanced to a large extent by simply reducing the distance between the source and the drain electrodes.^56^, ^69^, ^72^, ^73^, ^74^, ^75^ It should be noted that, when comparing the performances of photoconductor and phototransistor structured devices, it is essential to employ devices with comparable dimensions.

#### 2.1.4 Perovskite/Electrode Interface

Interfacial engineering is of essential importance for OIHPs‐based PDs in order to achieve outstanding output performance, especially at the perovskite/electrode interface.^76^, ^77^ The commonly employed electrodes include gold (Au), silver (Ag), copper (Cu), platinum (Pt), titanium (Ti), aluminum (Al), graphene, and indium tin oxide (ITO).^35^, ^78^, ^79^, ^80^, ^81^, ^82^ Different metals with varying Fermi levels will form Ohmic or Schottky contacts with OIHPs due to energy alignment, resulting in symmetrical and linear or asymmetrical and nonlinear *I*–*V* characteristics.^82^ The Schottky barriers may also derive from the surface state of OIHPs such as surface defects, vacancies, and adsorption besides inappropriate band alignment at the perovskite/electrode interface.^80^ Therefore, the optoelectronic performance OIHPs‐based PDs can be optimized by careful electrodes selection, refined OIHPs film fabrication, and delicate interface modification.^35^, ^79^, ^82^, ^83^, ^84^

### 2.2 External Factors

Besides the internal factors discussed above, external factors such as the testing conditions and environment exert a significant influence on the output performance of OIHPs‐based PDs.

#### 2.2.1 Testing Conditions

Since *R* is closely related to the incident light intensity (Equation (4) and Figure 2g), superhigh *R* values can thus be obtained when irradiated by extremely low light powers.^56^, ^69^, ^70^, ^71^, ^72^, ^75^, ^85^, ^86^, ^87^, ^88^, ^89^, ^90^ Other differences in testing conditions such as the bias voltages and the incident wavelengths will also affect the electrical performance of the devices. Therefore, these testing conditions as well as above‐mentioned channel width should be taken into consideration to more precisely and objectively evaluate and compare the output performances of different PDs.

#### 2.2.2 Environment

For practical applications, heat, light irradiation, water invasion, and oxygen erosion are the primary factors having a significant effect on the electrical performances of OIHPs‐based PDs because of the intrinsic instability of organic cations. The encapsulation techniques used in the photovoltaic industry can be adopted to prevent water and oxygen invasion. Common sealing polymer materials such as polymethyl methacrylate (PMMA), fluorous polymer (CYTOP), PS, and polylactic acid (PLA) can be used.^25^, ^50^, ^64^, ^80^, ^89^ Other strategies include designing materials with special micromorphologies (e.g., micro/NW, nanonet, nanoplates, etc.) and incorporating functional materials allowing PDs with long‐term stability.^53^, ^91^

## 3 OIHPs‐based PDs

### 3.1 Single Component OIHPs‐based PDs

#### 3.1.1 3D Thin‐Film Devices

**Figure 3:**
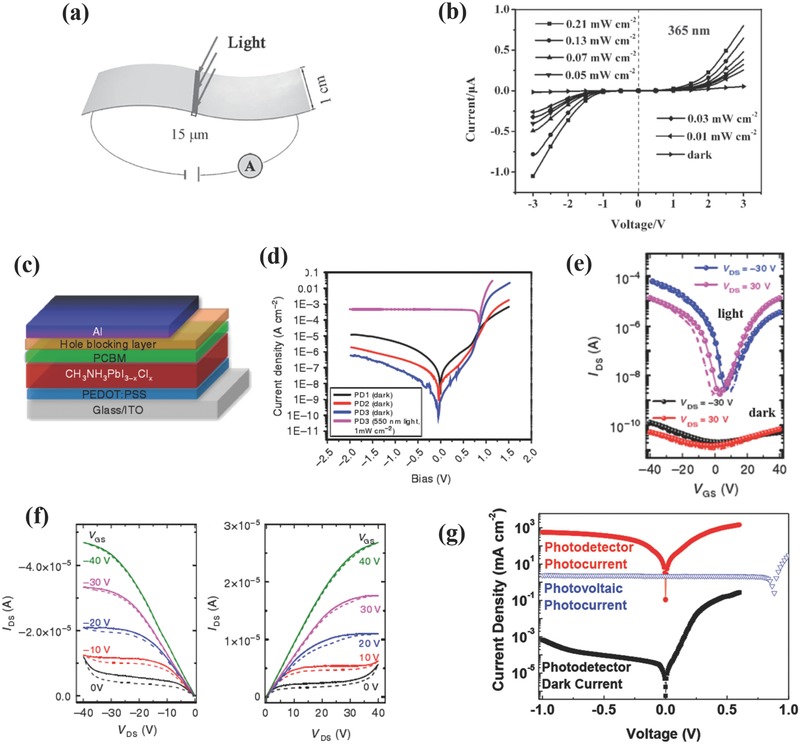
a) Device structure of flexible OIHPs‐based PDs. b) *I–V* curves of PDs at different light intensities and a fixed wavelength. a,b) Reproduced with permission.^80^ Copyright 2014, Wiley‐VCH. c) Device structure of perovskite PDs with SC configuration. d) *J–V* characteristics of the PDs without (PD1) and with HBLs (PD2 with BCP and PD3 with PFN). c,d) Reproduced with permission.^32^ Copyright 2014, Macmillan Publishers. e) Transfer curves of OIHPs‐based PDs measured in the dark and at a light illumination of 10 mW cm^−2^. f) Output characteristics of OIHPs‐based PDs at light illumination with gate voltages ranging from −40 to 40 V. e,f) Reproduced with permission.^25^ Copyright 2015, Macmillan Publishers. g) *J–V* characteristics of OIHPs‐based PDs measured at a light illumination of 10 mW cm^−2^ and at dark conditions. Reproduced with permission.^35^ Copyright 2015, Wiley‐VCH.

The first OIHPs‐based PDs was proposed by the Xie and co‐workers, as shown in **Figure** **3**a.^80^ The perovskite precursor solution was spin‐coated on a flexible polyethylene terephthalate (PET) substrate patterned with ITO electrodes. Figure 3b shows *I*–*V* curves measured at different light intensities and fixed wavelength (365 nm). Upon light illumination, the *I*_light_ increased drastically from a nearly insulating state at dark conditions and demonstrated asymmetrical and nonlinear characteristics resulted from the Schottky barrier at the perovskite/ITO interface. The devices showed excellent photoresponses with an *R* of 3.49 A W^−1^, an EQE of 1.19 × 10^3^%, an on/off ratio of 152 at 1 V and response time lower than 0.2 s, respectively. Decent performances (*R* of 0.0367 A W^−1^, EQE of 5.84%, on/off ratio of 6.7 at 2 V and response time < 0.1 s) were also obtained when illuminated with a 780 nm laser, thereby suggesting high reproducibility, broad‐band detection capability, and fast response speed for the devices. Nearly simultaneously, Yang and co‐workers fabricated PDs with a “glass/FTO/PEDOT: PSS/CH_3_NH_3_PbI_3−_ *_x_*Cl*_x_*/PCBM/HBL/Al” device architecture (see Figure 3c), in which conjugated polymers (BCP and PFN) were used as hole transporting layers (HTLs), respectively.^32^ As depicted in Figure 3d, PD2 and PD3 with BCP and PFN, respectively, demonstrated reduced dark currents at reverse bias compared with PD1 without HTLs. The calculated D* was high as 10^14^ Jones. These devices also showed fast response times (τ_rise_ of ≈180 µs, τ_decay_ of ≈160 ns), a noise current lower than 1 pA Hz^−1/2^, reduced hysteresis effect, and suppressed dark current characteristics originating from a precise interface modification. Although photovoltaic‐like OIHPs‐based PDs respond significantly faster than planar devices, the complicated interface engineering between OIHPs and other semiconductors, the multilayer stacking configuration, and the high cost of HTLs or electron transporting layers (ETLs) limit their practical commercialization to a certain extent.

Encouraged by these two pioneering works, a large amount of outstanding works have been reported in an attempt to further improve the output performances of OIHPs‐based PDs. Wu et al. have fabricated polycrystalline perovskite into phototransistors, and suggested that the thickness of perovskite is a crucial factor determining the performance of the device. Thus, excessively thin films cannot absorb sufficient light while too‐thick films will not allow light to penetrate the whole film, thereby avoiding effective gate tuning effect.^25^ They found that the optimum perovskite thickness was to be ≈ 100 nm and the corresponding phototransistor displayed ambipolar *I*–*V* transfer characteristics at drain voltage of – 30 and 30 V, as shown in Figure 3e. These devices exhibited a noticeable photosensitivity under light (0. 1 mA) and dark (0.5 nA) conditions with high on/off ratios reaching 3.32 × 10^4^ and 8.76 × 10^3^ for p‐type and n‐type transport, respectively. The extracted hole and electron mobilities approached 0.18 and 0.17 cm^2^ V^−1^ s^−1^, respectively. Figure 3f is the output characteristics of PDs with gate voltages varying from −40 to 40 V under an illumination of 10 mW cm^−2^. The devices presented diode‐like ambipolar transport at low *V*_gs_ (0 V) and transistor‐like unipolar characteristics at high *V*_gs_ (−40 V), with calculated *R* as high as 320 A W^−1^. As another type of PDs, the photodiode configuration was commonly adopted in many studies because of its advantages in low driving voltage, fast response time and high photoconductive gain characteristics. For example, the Huang and co‐workers have prepared perovskite materials by two‐step spin‐coating method and designed the corresponding PDs with a photovoltaic‐like “ITO/perovskite/TDP‐Si_2_/MoO_3_/Ag” structure.^35^ These devices showed a Schottky‐rectifying photodiode behavior at dark as a result of large energy barriers for electron and hole injection and an Ohmic photoconducting response under light illumination originating from the abundant trapped holes induced band bending between the perovskite and the MoO_3_/Ag electrode (see Figure 3g). Further theoretical calculations and photothermal induced resonance technique have proved that the decomposition of perovskite has left abundant Pb^2+^ clusters on the surface, inducing hole traps in the devices. Thus the maximum *G* reached 489 at −1 V and R was as high as 242 A W^−1^ at 740 nm, with ultrafast response times (τ_rise_ of ≈ 10 µs, τ_decay_ of ≈ 5.7 µs), a reasonable LDR of 85 dB, and a low NEP at 0.18 pW Hz^−1/2^. Soon afterward, by delicate interface engineering and morphology optimization, the same group has realized sub 1 pW cm^−2^ light detection, which matched with the calculated NEP value for the first time.^92^ In this regard, a double fullerene layer (PCBM/C_60_) was added to passivate charge traps and suppress dark current, and the wettability of ETLs (PEDOT:PSS and OTPD) was utilized to control the grain size of the perovskites. These as‐prepared devices exhibited low noise of 16 fA Hz^−1/2^ at −0.1 V, large LDR of 94 dB, and ultrafast response time of 120 ns.

#### 3.1.2 3D Single Crystalline Devices

**Figure 4:**
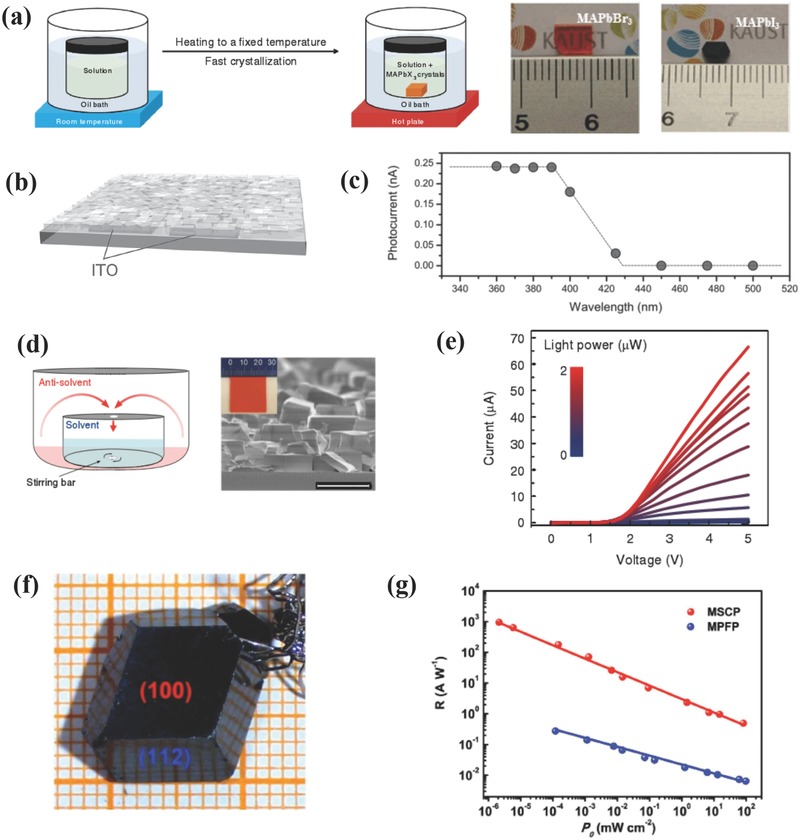
a) Schematic diagram of the ITC process (left) and pictures of the as‐synthesized MAPbBr_3_ and MAPbI_3_ single crystals (right). Reproduced with permission.^94^ Copyright 2015, Macmillan Publishers Limited. b) Device structure and c) spectral photocurrent of MAPbCl_3_ single crystal‐based PDs. b,c) Reproduced with permission.^95^ Copyright 2016, Wiley‐VCH. d) Schematic diagram of the AVC technique (left) and SEM image of the as‐synthesized MAPbBr_3_ single crystals (right, scale bar = 50 µm); e) *I*–*V* characteristics measured at different light intensities. d,e) Reproduced with permission.^97^ Copyright 2015, Macmillan Publishers. f) Photos of MAPbI_3_ bulk single crystals fabricated by the BSSG technique. g) Responsivity versus incident‐light density for MAPbI_3_ single crystal PDs (MSCP) and their polycrystalline counterpart (MPFP). f,g) Reproduced with permission.^100^ Copyright 2015, Macmillan Publishers.

Besides exploiting the full potential of OIHPs in optoelectronics, it is also very important to investigate the fundamental properties behind them. Therefore, single crystal materials have stood out because of their unique properties (e.g., high purity, few grain boundaries, and enhanced stability toward heat and moisture) as compared to polycrystalline counterparts, which make them perfect for unveiling the inherent photophysical properties of perovskites.^93^ Numerous facile approaches for synthesizing OIHPs single crystals have been developed by researchers, and the most commonly used approach is inverse temperature crystallization (ITC).^94^ Contrary to the common law of solubility that solutes have a higher solubility in solvents at elevated temperatures, MAPbX_3_ exhibits a fascinating inverse solubility trend in some solvents, thereby enabling fast growth of large‐sized and high‐quality single crystals. As depicted in **Figure** **4**a, the precursor solutions were heated from room temperature and subsequently kept at critical temperatures (80 °C for MAPbBr_3_ and 110 °C for MAPbI_3_). At these conditions, the nuclei took shape and grew up to several millimeters in just a few hours with a high growth rate (maximum 38 mm^3^ h^−1^). Both structural and morphological characterizations have confirmed that MAPbX_3_ owned the high‐quality single crystalline nature of with large grain sizes and few grain boundaries. The transport properties were also conducted in detail, presenting high charge carrier mobilities of 24.0 and 67.2 cm^2^ V^−1^ s^−1^, low trap densities (*n*_trap_) of 3 × 10^10^ and 1.4 × 10^10^ cm^−3^, long diffusion lengths of 4.3 and 10.0 µm, fast‐component excitons lifetimes (τ) of 28 ± 5 and 18 ± 6 ns and slow‐component lifetimes of 300 ± 26 and 570 ± 69 ns for MAPbBr_3_ and MAPbI_3_, respectively. As for MAPbCl_3_, large‐sized single crystals were also prepared by ITC to achieve visible‐blind UV‐sensitive detection in virtue of their wide band gap properties. The Sargent and co‐workers have introduced stirring force during ITC technique to increase nucleation rate and obtained compact and interconnected MAPbCl_3_ single crystals with micrometer dimensions on ITO substrate (see Figure 4b).^95^ The electrical and transport properties were investigated carefully and demonstrated photoluminescence (PL) decay time of 4 ns (fast component) and 31 ns (slow component), high hole mobility of 26 cm^2^ V^−1^ s^−1^ and low hole density of 10^10^ cm^−3^, outperforming the thin film counterpart. PDs based on this material exhibited rectifying *I*–*V* characteristics at dark but an Ohmic behavior under light illumination with different light intensities, and the as‐obtained *R* reached 18 A W^−1^ at 4 nW, with G approached 100, D* yielded 10^12^ Jones, and limited fall time was 1 ms. Figure 4c shows the spectral photocurrents of the PDs, with the “threshold” wavelength being well positioned at 420 nm and matching with PL and UV–vis absorption spectrum, thereby making this device particularly useful for detecting UV light. More recently, Kuang et al. have improved this approach by developing an advanced space‐limited ITC technique, in which a narrow space was technically designed to constrain the lateral growth of OIHPs.^93^ The resulting MAPbBr_3_ with an area of 120 cm^2^ and thickness of 0.1 – 0.8 mm was grown on substrates patterned with fluorine‐doped tin oxide (FTO). This material has demonstrated comparable or superior optical and transport characteristics compared with previously reported materials.^96^ Therefore, the corresponding PDs showed excellent photodetection capabilities with broad linear response versus incident light intensities ranging from 10^−4^ to 10^2^ mW cm^−2^, reasonable response time of 0.12 ms with a thickness of 0.1 mm, wavelength selectivity of 61.3 and 3 dB cut‐off frequency (*f*_3 dB_) of 100 kHz at −1 V bias.

Additional facile techniques used to synthesize OIHPs single crystals include antisolvent vapor‐assisted crystallization (AVC) and top‐seeded solution growth (TSSG). Figure 4d shows the schematic diagram of a typical AVC process, in which dichloromethane (DCM) as an antisolvent is gradually diffused into solvent (*N*,*N*‐dimethylformamide (DMF)) containing MABr and PbBr_2_ to induce nucleation. These nuclei progressively grow by absorbing the precursors in the solvent to form crack‐free and well‐defined MAPbBr_3_ single crystals of several millimeters in size (see Figure 4d, right).^97^ Moreover, these single crystals exhibited exceptional electric and transport properties, comparable to those of materials prepared by ITC.^98^ However, AVC technique was found to favor the formation of free‐standing 3D perovskites but it was unsuccessfully integrated on substrates, which limits its application in optoelectronics. To overcome this issue, an additional stirring force was introduced to perturb the normal AVC process, thereby increasing the number of nucleation sites and obtaining 2D, micrometer‐sized continuous perovskites on an ITO substrate. The high‐quality nature and outstanding transport properties of MAPbBr_3_ single crystals have enabled superior optoelectronic properties. As shown in Figure 4e, when the *I*_light_ values were measured at the microampere (µA) scale and low light intensities, a rectifying *I*–*V* behavior resulting from metal‐semiconductor‐metal junction was demonstrated. A high *R* value exceeding 4 × 10^3^ A W^−1^ was calculated, with *G* surpassing 10^3^, decay time of 25 µs and ultrahigh D* approaching 10^13^ Jones. TSSG can be also used to prepare millimeter‐sized perovskites single crystals by employing temperature gradient. The basic principle of TSSG can be explained as follows: The hot bottom precursor solution is maintained saturated with the presence of small‐sized MAPbI_3_ single crystals while the cool top solution is supersaturated. Subsequently, large‐sized MAPbI_3_ single crystals were gradually grown on the silicon substrate driven by the low temperature gradient between the top and the bottom solutions. The as‐prepared MAPbI_3_ single crystals showed an average size of 3.3 mm with the largest crystal extending to 10 mm, and these high‐quality materials possessed excellent optical, electrical, and transport properties with a diffusion length exceeding 175 µm.^99^ Sun et al. have prepared (100)‐faceted MAPbI_3_ single crystals with millimeter‐sized dimensions by using a bottom‐seeded solution growth method (see Figure 4f).^100^ The PDs based on these bulk single crystals showed maximum *R* of 953 A W^−1^, EQE of 2.22 × 10^5^%, fast rise, and decay times of 74 and 58 µs as well as enhanced stability (only 6% drop of photocurrent after 40 d in air). As depicted in Figure 4f, the bulk MAPbI_3_ single crystals‐based PDs showed a lower detectable irradiance density at 2.12 nW cm^−2^ than their polycrystalline counterparts at 120 nW cm^−2^, further revealing the advantage of this synthesis technique. AVC and TSSG are expected to be more broadly used for scale‐up fabrication and commercialization if faster growth rates of perovskite single crystals can be technically achieved. **Table** **1** summarizes the optoelectronic performance of some PDs based on 3D OIHPs thin‐film or single crystals.

**Table 1:** Optoelectronic performances of 3D thin‐film and single‐crystalline perovskites‐based PDs

Device architecture	*R* [A W^−1^]	D* × 10^12^ [Jones]	EQE [%]	On/off ratio	*G* [×10^3^]	LDR [dB]	NEP [pW cm^−2^]	*t*_rise_/*t*_decay_ [ms]	Ref.
Pt/MAPbCl_3_/Ti/Au	0.0469	0.012	–	–	–	–	–	24/62	145
FTO/ETL/ MAPbI_3_/HTL/Au	620	–	2.4 × 10^5^	–	2.4	–	–	100–200	146
ITO/HTL/perovskite/ETL/Al	–	7.4	≈90	–	–	94	0.6	1.2 × 10^−4^	92
ITO/perovskite/MoO_3_/Ag	242	–	–	–	0.489	85	0.18	0.01/0.006	35
Au/perovskite/Au	320	–	–	10^4^	0.01–0.1	–	–	<0.01	25
ITO/HTL/perovskite/ETL/LiF/Al	–	>1	50–70	–	–	170	200	0.005/0.003	147
ITO/TiO_2_/perovskite/P3HT/MoO_3_/Ag	0.339	4.8	84	–	–	100	–	≈10^−4^	148
ITO/perovskite/ITO	4 × 10^3^	>10	–	–	>10	–	–	0.025	97
ITO/ MAPbCl_3_/ITO	18	1	–	–	0.1	–	–	0.001	95
ITO/HTL/perovskite/ETL/Al	0.321	–	60	–	–	84	–	0.004/0.003	78
Pt/perovskite/Pt	–	0.13	–	10^4^	–	–	–	90/20	81
ITO/perovskite/ITO	1640	10	10^5^	–	2.5	70	–	0.03/0.02	149
Au/perovskite/Au	2.36	1.5	639	>10^3^	–	–	–	<4	150
Au/perovskite/Au	10.33	–	–	10^5^	–	–	–	0.02/0.01	62
Au/ MAPbI_3_/Au	953	–	2.2 × 10^5^	224	–	76	–	0.07/0.06	100
ITO/ MAPbI_3_/ITO	3.49	–	1.2 × 10^3^	324	–	–	–	<200	80
ITO/HTL/perovskite/ETL/Al	–	100	–	–	–	100	4.6	6 × 10^−4^	32
Au/perovskite/Au	1.9 × 10^4^	–	4.9 × 10^6^	–	53	–	–	<450	151
Au/ MAPbI_3_/Au	7.92			130				<200	152

#### 3.1.3 2D Nanoplate Devices

Recent years have witnessed a skyrocketing progress in 2D materials family, best exemplified by graphene, transition‐metal dichalcogenides (TMDs), hexagonal boron nitride (h‐BN), and black phosphorous.^101^, ^102^, ^103^, ^104^, ^105^, ^106^ Compared with 3D large‐sized materials, 2D materials possess unique properties that have greatly attracted the attention of the scientific community: (i) high specific surface area; (ii) weak van der Waals interlayer bonding and high mechanical strength applicable to flexible devices; and (iii) high PL quantum yield, strong quantum confinement, and tunable optical band gaps desired for optoelectronics. Therefore, it is intriguing to reduce the thickness of OIHPs down to a few unit cells and investigate the effect of this reduced dimensionality on the intrinsic properties. Currently, there are two kinds of “2D” OIHPs nanoplates namely, non‐van der Waals‐type (derived from corresponding 3D framework by cutting down the thickness to single or few unit cells) and van der Waals‐type built by inserting long organic cations in the “A” position to block the interaction of inorganic [BX_6_]^4−^ bilayers. Next, we will introduce the OIHPs nanoplates and their corresponding PDs.^56^

**Figure 5:**
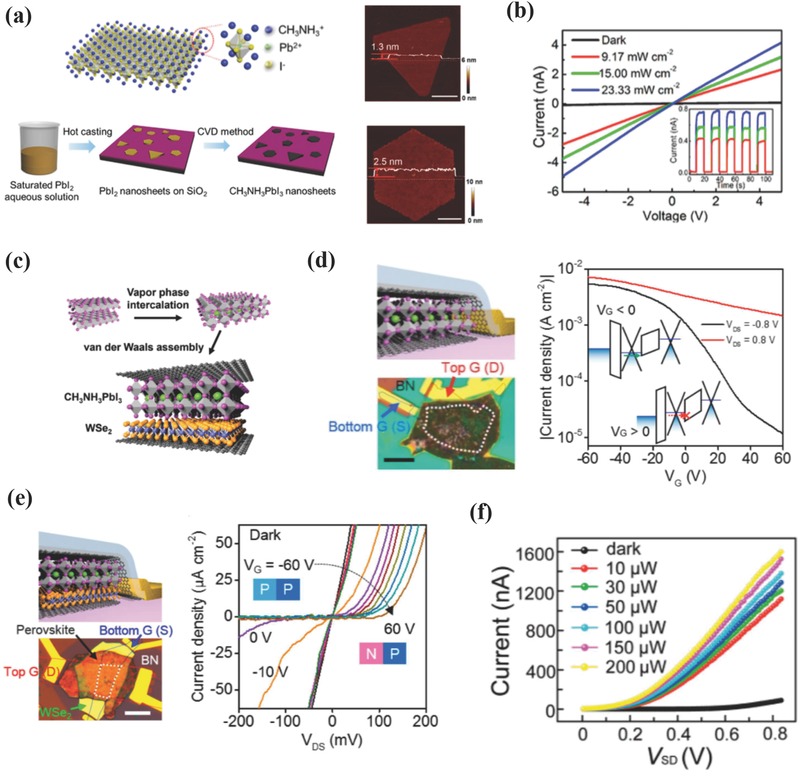
a) Crystalline structure of a 2D MAPbI_3_ with single‐unit‐cell thick (left, top), schematic diagram of a 2D MAPbI_3_ preparation process (left, bottom), and the AFM topography images of 2D MAPbI_3_ with triangular (right, top) and hexagonal morphologies (right, bottom). Scale bar: 2 µm. b) *I*–*V* characteristics of the 2D MAPbI_3_‐based PDs measured at different light intensities. a,b) Reproduced with permission.^56^ Copyright 2016, ACS. c) The Schematic diagram of the perovskite conversion from PbI_2_ flakes into MAPbI_3_ under MAI vapor atmosphere. d) Schematic and optical images of a BN‐protected GPG device (left) and transfer curves of the devices measured at positive (0.8 V) and negative (−0.8 V) drain biases at dark and 77 K (right). e) Schematic and optical images of BN‐protected GWPG devices (left) and *I*_DS_–*V*_DS_ characteristics measured at dark with different gate biases (right). c–e) Reproduced with permission.^72^ Copyright 2015, ACS; f) *I*–*V* curves of (C_4_H_9_NH_3_)_2_PbBr_4_‐based PDs at different incident powers. Reproduced with permission.^53^ Copyright 2016, ACS.

With regard to non‐van der Waals‐type OIHPs, Bao and colleagues have prepared CH_3_NH_3_PbX_3_ nanosheets as thin as a single unit cell via a two‐step solution and vapor combined method.^56^ As shown in **Figure** **5**a, a saturated PbX_2_ hot solution was cast on a SiO_2_/Si substrate, and the vaporized CH_3_NH_3_X was subsequently intercalated into the crystal lattice of PbX_2_ at an elevated temperature. The mechanism behind the formation of this 2D structure involves the preferential growth along the in‐plane direction induced by a lower surface energy. The as‐synthesized OIHPs nanoplates showed varying thickness of 1.3–13 nm (corresponding to 1–10 unit cells), with the other two dimensions extending to several micrometers. The PDs based on these materials demonstrated rather low *I*_dark_ and linear *I*–*V* curves (10^−9^ A) under light illumination (Figure 5b). Thus the *I*_light_/*I*_dark_ ratio of the devices reached ≈10^2^, while *R* was as high as 22 A W^−1^ at 1 V bias irradiated by a weak laser (*P* < 10 nW) and response times was lower than 40 ms. CH_3_NH_3_PbI_3_ nanoflakes with a thickness of 43.2 nm were prepared by mechanically exfoliating PbI_2_ flakes and subsequent methylammonium iodide (MAI) vapor phase intercalation (see Figure 5c).^72^ The as‐fabricated “graphene/perovskite/graphene” (GPG) PDs demonstrated appealing properties, with an *R* of ≈ 950 A W^−1^, a *G* of 2.2 × 10^3^, rise and decay times of 22 and 37 ms, respectively. An additional BN layer was subsequently transferred to protect the vulnerable GPG architecture from ambient conditions, and the devices showed no major photocurrent decay after 210 d. Figure 5d shows the transfer curves of BN‐protected GPG devices measured at dark and 77 K. Positive source‐drain bias (0.8 V) led to low on/off ratio (≈5) while negative bias (−0.8 V) resulted in an on/off ratio of ≈500. These results can be ascribed to the gate tunability of the graphene work function and the Schottky barriers for hole injections at the bottom‐graphene/perovskite interface. *I*_ds_−*V*_ds_ measurement were also conducted and confirmed that gate tuning effect was originated from the work function modulation for top and bottom graphene, which caused a built‐in electric field responsible for charge carriers dissociation and transport even at zero bias. To further investigate the gate tunability, tungsten diselenide (WSe_2_) was inserted to form “GWPG” heterostructured devices. This kind of devices presented an enhanced on/off ratio at ≈10^6^ derived from the gate tuning effect of the Schottky barriers at the bottom‐graphene/WSe_2_ and WSe_2_/perovskite interfaces. As depicted in Figure 5e, almost linear *I*–*V* characteristics were observed at gate voltages ranging from −60 to −20 V, and a diode‐like rectifying behavior was observed with the gate bias increasing to positive values as a result of the ambipolar nature of WSe_2_. The unique van der Waals GPG and GWPG device stacking structures have minutely elaborated the underlying physical properties behind the electrical behaviors of these devices, and this has favored novel optoelectronic designs and fabrication methods.

With regard to van der Waals type OIHPs, the Ruddlesden–Popper type perovskite is chosen here to illustrate the intrinsic properties and photoelectronic applications of these materials. (PEA)_2_(MA)*_n_*_−1_Pb*_n_*I_3_ *_n_*_+1_, (BA)_2_(MA)*_n_*_–1_Pb*_n_*I_3_ *_n_*_+1_, and other 2D layer‐structured OIHPs have been intensively investigated and applied to photovoltaic fields with promising PCEs.^8^, ^107^, ^108^, ^109^, ^110^, ^111^, ^112^, ^113^, ^114^ A p‐type (C_6_H_9_C_2_H_4_NH_3_)_2_PbI_4_ perovskite was studied to reveal the photophysics behind this van der Waals structure, and the Wannier–Mott excitons were suggested to strongly contribute to the *I*_light_ despite their greater binding energy (*E*_b_) at room temperature.^115^ In addition, the *I*_light_ was enhanced by 100 times and the *G* value increased by 10 folds upon incorporating ETL and HTL, confirming this material as a promising candidate for optoelectronics. Atomically thin (C_4_H_9_NH_3_)_2_PbBr_4_ has been synthesized via a ternary cosolvent method, which exhibited a unique structural relaxation behavior, strong PL, and color and composition tuning characteristics.^52^, ^107^ However, the presence of insulating organic cations can inhibit out‐of‐plane charge transport, leading to poor conducting performances and hindering practical applications. Bearing this in mind, Peng et al. fabricated (C_4_H_9_NH_3_)_2_PbBr_4_‐based PDs by integrating this material with a large‐area monolayer of graphene as a protective layer.^53^ As illustrated in Figure 5f, the photoresponse *I*–*V* curves showed a relatively low *I*_dark_ (10^−10^ A) and increasing *I*_light_ upon enhancing the incident powers. The calculated *I*_light_/*I*_dark_ ratio was 10^3^ at 5 V and 10 µW, and *R* was as high as 2.1 × 10^3^ A W^−1^. The introduction of graphene not only protected the perovskite nanoplates from moisture and polar solvents (e.g., acetone) attack, but also drastically increased the photoresponsivity, which is essential for the performances of PDs.

#### 3.1.4 1D NW Devices

1D NW materials (e.g., carbon nanotubes (CNTs), metal NWs, metal oxides NWs, and polymer NWs) have been deeply investigated in the past decades because of their unique mechanical and optoelectronic properties: (i) 1D geometry with excellent resilience to stress that benefits the manufacture of flexible devices; and (ii) large surface‐to‐volume ratio that leads to prolonged charge carrier lifetimes and reduced carrier recombination.^116^, ^117^ It is hence desirable to fabricate OIHPs‐based NWs (PNWs) to explore their potential applications, especially in optoelectronics.^118^

**Figure 6:**
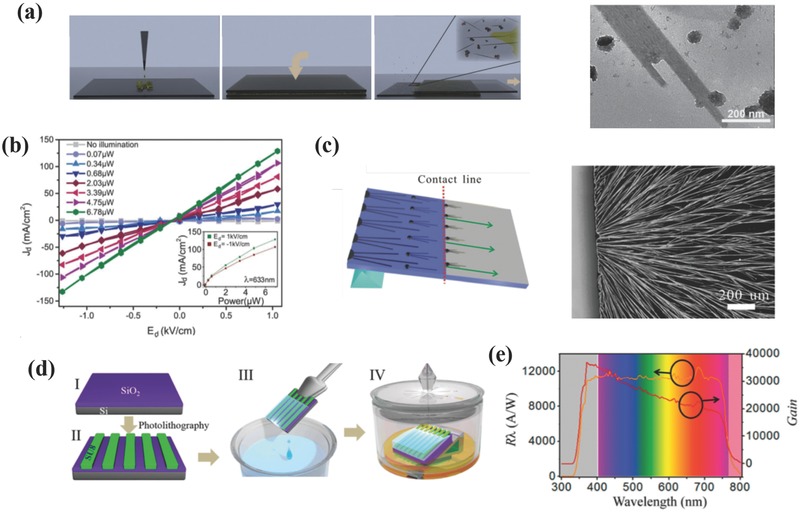
a) Schematic illustration of the PNWs synthesis by a low‐temperature solution‐processed slip‐coating method (left) and TEM image of the as‐synthesized PNWs (right). b) *I*–*V* characteristics of the PNWs‐based PDs at dark and various light intensities. a,b) Reproduced with permission.^119^ Copyright 2014, ACS. c) Schematic illustration of the modified EISA technique (left) and optical images of the as‐synthesized PNWs grown from the edge of the substrate (right). Reproduced with permission.^59^ Copyright 2015, Royal Society of Chemistry (RSC). d) Schematic illustration of the FGAVC technique. e) Photoresponsivity and photoconductive gain against different light wavelengths. d,e) Reproduced with permission.^121^ Copyright 2017, ACS.

Recently, several groups have reported on synthesis techniques for PNWs and explored the potential applications in PDs. Horváth et al. have synthesized PNWs via a low‐temperature crystallization technique by using a simple and low‐cost slip coating method.^119^ As illustrated in **Figure** **6**a (left), the saturated perovskite precursor solution was dropped on the glass slide and covered with another glass to squeeze out the excess solution. The upper glass slide was subsequently removed gently from one side and the uniform perovskite precursor solution gradually evaporated with an instant color change from yellow to brown‐red. The as‐synthesized PNWs showed an average width ranging from 50 to 200 nm with a length up to 16 µm and height ranging from 9 to 90 nm (see Figure 6a, right). The PDs based on these PNWs exhibited moderate photoresponses at different light intensities (see Figure 6b), with an *R* value at 5 mA W^−1^ and response time lower than 500 µs. However, the random distribution of PNWs induced by the gradual evaporation of DMF solvent may result in low utilization and poor performances because of the mismatch between the PNWs and the prepatterned electrodes. Later, a modified evaporation induced self‐assembly (EISA) method was used to prepare high‐ordered PNWs, as illustrated in Figure 6c.^59^ The perovskite precursor solution was dropped on a tilted substrate (< 15°), and then the large‐area aligned PNWs were gradually formed driven by solvent evaporation and gravitational force. This methodology resulted in PNWs with an average width of 500 nm and length extending to 800 µm. The PDs fabricated with these materials showed R as high as 1.32 A W^−1^, D* of 2.5 × 10^12^ Jones, *I*_light_/*I*_dark_ ratio of 23 and response time of 0.3 ms. These values are superior than those of their randomly distributed PNWs counterparts. However, it is noteworthy that *I*_light_/*I*_dark_ ratio is still very low compared with polycrystalline OIHPs‐based PDs, hampering their future applications in optoelectronics to a certain extent.^59^ Lately, the Tang and co‐workers have also used this method to prepare single‐crystalline PNWs with surface defects passivated via an oleic acid (OA) toluene soaking. This methodology allowed materials with lower trap densities, higher carrier lifetime, and enhanced stability and photosensitivity characteristics.^57^ The corresponding PDs exhibited enhanced *I*_light_/*I*_dark_ ratio of 4 × 10^3^ with a response time lower than 0.1 ms, an *R* of 4.95 A W^−1^, and a D* of 2 × 10^13^ Jones, realizing strong polarized light detection for the first time. Besides the commonly used EISA technique, template‐assisted solvatomorph graphoepitaxy has been utilized to control the growth of PNWs involving the “solvent evaporation–supersaturation–crystallization” process.^90^ When irradiated with an ultralow light power of 2.51 pW, the devices exhibited a high R value of 6 × 10^6^ A W^−1^ and a slow response time of 2.5 s. A self‐templated‐directed synthesis technique was introduced by Zhang and co‐workers by reacting the lead‐containing NW precursor with CH_3_NH_3_Br and HBr.^58^ This facile approach led to single PNWs based devices with reasonable sensitivities (an on/off ratio of 61.9 and rise and decay times of 0.12 and 0.086 s, respectively) and high stability under ambient conditions. To solve the integration issue of randomly distributed PNWs, roll‐to‐roll (R2R) microgravure printing and doctor blading techniques have been preferred because these techniques are more applicable to large‐area and low‐cost integrated electronics on rigid or flexible substrates.^120^ More recently, a facile two‐step method called fluid‐guided antisolvent vapor‐assisted crystallization has been developed for synthesizing single‐crystalline PNWs arrays.^121^ As depicted in Figure 6d, aligned SU‐8 photoresist stripes were fabricated on SiO_2_/Si substrate by photolithography to act as a template. These stripes were subsequently dipped into the perovskite precursor solutions for a few seconds and placed on a titled glass (< 5°) sealed under CH_2_Cl_2_ vapor. The as‐synthesized PNWs exhibited a width of 400 nm and an ultrasmooth surface roughness of 1 nm, thereby revealing few defects and superior transport properties with a diffusion length of 41 µm. The corresponding devices demonstrated a maximum *R* of 1.26 × 10^4^ A W^−1^ and a high *G* value of 10^4^ in the visible spectrum (see Figure 6e), together with short rise and decay times of 0.34 and 0.42 µs, respectively, an NEP of 2.83 × 10^−12^ W Hz^−1/2^, a D* of 1.73 × 10^11^ Jones, an *I*_light_/*I*_dark_ ratio of 10^2^, an LDR of 150 dB, and enhanced air stability. Moreover, the band gap tunability of perovskite was utilized to synthesize a series of MAPb(*I*_1−_ *_x_*Br*_x_*)_3_ (*x* = 0–0.4) for integrated specific wavelength detection.

These advantages of PNWs have inspired tremendous efforts for developing various facile synthesis techniques and investigating the growth mechanisms behind them. The corresponding PDs exhibited superior optoelectronic performances, which are partly summarized in **Table** **2**.

**Table 2:** Optoelectronic performances of 1D NW peorvskites‐based PDs

Device architecture	Length [µm]/diameter [nm]	*R* [A W^−1^]	D* × 10^12^ [Jones]	On/off ratio	*t*_rise_/*t*_decay_ [ms]	Ref.
Pt/MAPbI_3_/Gra./Pt	0.93/68	6 × 10^6^	–	–	2 × 10^3^/1 × 10^4^	90
Au/ MAPbI_3_/Au	2 × 10^3^/100	4.95	20	4 × 10^3^	<0.1	57
Au/MAPbBr_3_/Au	10/200	–	–	61.9	120/86	58
Au/MAPbI_3_/Au	800/500	1.3	2.5	23	0.2/0.3	59
Pt/MAPbI_3_/Pt	10/50	5 × 10^−3^	–	300	0.35/0.25	119
Au/Cs*_x_*(MA)_1−_ *_x_*PbI_3_/Au	10^2^/300	23	0.25	4	10/20	153
Au/MAPbI_3_/Au	–/10^2^	–	–	13	120/210	118
Au/MAPbI_3_/Au	–/10^2^	1.3 × 10^4^	0.17	10^2^	3.4 × 10^−4^/4.2 × 10^−4^	121
Au/MAPbI_3_/Au	1.5 × 10^4^/10^2^	5.2 × 10^−3^	–	100	–	120
Au/MAPbI_3_/Au	10^2^/10^2^	0.1	1	300	0.3/0.4	141

#### 3.1.5 0D Nanocrystal Devices

When 3D materials are size‐decreased to nanometric scale, a remarkable size effect predominates, bringing about many striking properties such as size‐dependent optical band gaps, wide excitation over a narrow emission spectrum, large Stokes shift, and high luminescent efficiency, which are precisely the properties desirable for LEDs and other photodevices.^23^ This kind of materials are called quantum dots or nanocrystals (NCs). OIHPs‐based NCs (PNCs) are therefore expected to display unusual properties that can be potentially applied in optoelectronics such as PDs.

**Figure 7:**
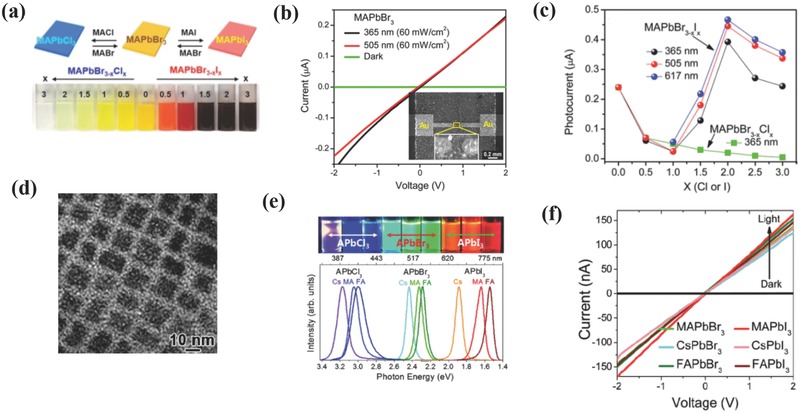
a) Reversible anion exchange reaction of MAPbX_3_ NCs (top) and photos of the mixed halide MAPbX_3_ colloidal solutions (bottom). b) *I–V* characteristics of MAPbBr_3_ measured at 365 and 505 nm laser and dark conditions. c) Photocurrents of the mixed halide MAPbX_3_ as a function of *x* measured at 365, 505, and 617 nm. a–c) Reproduced with permission.^60^ Copyright 2015, ACS. d) HRTEM image of MAPbBr_3_ NCs. e) Photos of mixed APbX_3_ colloidal NCs irradiated by UV lamp (top) and PL spectra of the mixed APbX_3_ colloidal NCs, where A = MA^+^ and Cs^+^ and X = Cl^−^, Br^−^, and I^−^ (bottom). f) *I–V* characteristics of APbX_3_ under 365 nm laser and at dark, where A = MA^+^, Cs^+^, and FA^+^ and X = Cl^−^ and I^−^. d–f) Reproduced with permission.^61^ Copyright 2016, RSC.

Plate‐like MAPbBr_3_ NCs and their derivatives have been successfully synthesized with an average thickness of 5 nm and lateral lengths of 70 nm.^60^ Octylamine (OAm) was used as a capping agent to precisely control the thickness of these materials. Reversible halide exchange reactions were conducted to tune the chemical composition of MAPbBr_3_ yielding a series of mixed halide MAPbBr_3−_ *_x_*Cl*_x_* and MAPbBr_3−_ *_x_*I*_x_* compounds. As shown in **Figure** **7**a, this process demonstrated a distinguished color change and a full‐range optical band gap tuning over a wide absorption spectrum (1.6 – 3 eV). The PDs fabricated on these materials showed different photoresponses depending on the type of halides. Thus, in the case of pure CH_3_NH_3_PbBr_3_ NCs‐based PDs (see Figure 7b), the *I*–*V* curves were almost linear within −2 to 2 V, with an *I*_dark_ as low as 1 pA and an *I*_light_ (Δ*I* = *I*_ph_ – *I*_dark_) of 0.24 µA at 365 and 505 nm laser irradiation, respectively. In the case of mixed halide PNCs‐based PDs (see Figure 7c), Δ*I* decreased with increasing *x* for Cl‐rich CH_3_NH_3_PbBr_3−_ *_x_*Cl*_x_*, while I‐rich MAPbBr_3−_ *_x_*I*_x_* showed a maximum Δ*I* at *x* = 2. These results were highly consistent with the corresponding PL decay times, and were rooted in crystalline structure nature (i.e., the tetragonal phase displayed higher photoconversion efficiencies than that of the quasi‐cubic phase). Thus, the best performance of MAPbBrI_2_ was mainly originated from its pure tetragonal phase. Guided by the above study, later the same group reported for the first time a simpler and more convenient ultrasound‐assisted synthesis for colloidal APbX_3_ NCs, where ultrasonic irradiation assisted the dissolution of precursor and accelerated the reaction speed.^61^ The as‐formed rectangular MAPbBr_3_ NCs exhibited an average size of 10 nm with an average thickness lower than 5 nm, as shown in Figure 7d. As depicted in Figure 7e, a series of mixed halide PNCs showed a band gap tuning over a wide range of the PL spectra by changing the composition of the cations and halides. The corresponding PDs demonstrated similar photosensitivity with an *I*_dark_ of 1 pA and Δ*I* of 150 nA at 2 V bias voltage and under 365 nm illumination (see Figure 7f).

Currently, studies on PNCs‐based PDs are quite limited mainly because continuous and smooth thin films are difficult to obtain in the presence of capping ligands. Worse still, the quantum effects would disappear upon aggregation and growth of PNCs, and these materials are vulnerable to water and other polar solvents. Therefore, a significantly higher attention has been paid to the synthesis techniques and fundamental physics of PNCs. For example, Julia Pérez‐Prieto et al. have first synthesized solution‐processed MAPbBr_3_ nanoparticles by using medium‐sized organic chains to allow for uniform dispersion in organic solvents.^122^ The as‐prepared high‐quality nanoparticles exhibited a high quantum yield, which might be advantageous for optoelectronic devices. Afterward, Zhang and co‐workers reported on the formation of PNCs with varying sizes and high PL quantum yields.^123^ During the synthesis process, (3‐aminopropyl)triethoxysilane (APTES) and NH_2_‐POSS were used as branched capping ligands. In particular, the APTES‐capped PNCs demonstrated greater stability in protic solvents as compared to straight ligands because of their steric hindrance and hydrolysis propensity. Apart from colloidal synthesis, a template‐assisted approach has been studied by the Yamauchi and co‐workers to synthesize PNCs.^124^ They used highly ordered mesoporous silica as a template to precisely control the growth of PNCs, allowing the mutual separation them of PNCs, and improving their stability. By controlling the pore size of the template, PL shift and lifetime changes were directly observed, indicating that nonirradiative recombination pathways were introduced by localized surface states. Long‐term stability has become a major concern when considering the practical applications. Therefore, all inorganic PNCs have been gradually studied owing to their superior stability against oxygen and moisture than OIHPs counterparts, with the rest of optoelectronic performances being nearly comparable.^30^ With proper film treating and formation methods in place, high responsivity and fast response times of all inorganic PNCs based PDs can be successfully obtained, expanding the scope of the photodetection field.

### 3.2 Bilayer‐Structured OIHPs‐based PDs

In order to further improve the photoresponse, carrier mobility, and photosensitivity of planar‐structured photoconductors or phototransistors, a bilayer configuration involving gating effect has been adopted.^69^ In the following section, we will elucidate the device architectures, sensing mechanisms, and optoelectronic performance of bilayer‐structured perovskite/inorganic semiconductor and perovskite/organic semiconductor‐based PDs.

#### 3.2.1 Perovskite/Inorganic Semiconductor Bilayer Devices

**Figure 8:**
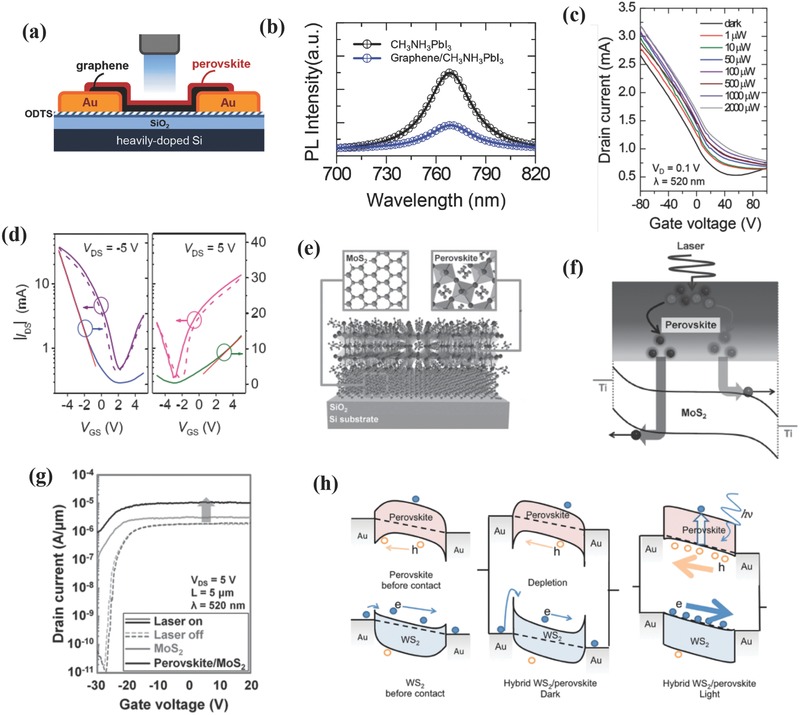
a) Schematic device architecture of hybrid perovskite–graphene PDs. b) PL spectra of pristine perovskite and hybrid perovskite–graphene excited by 532 nm laser. c) Transfer curves of hybrid perovskite–graphene PDs measured at different light intensities and at *V*_D_ of 0.1 V. a–c) Reproduced with permission.^70^ Copyright 2014, Wiley‐VCH. d) Transfer characteristics of hybrid perovskite/SWNTs PDs operating under light. Reproduced with permission.^125^ Copyright 2017, Wiley‐VCH. e) Schematic device architecture of hybrid perovskite–MoS_2_ PDs. f) Energy band diagram of hybrid perovskite–MoS_2_ PDs irradiated by laser. g) Transfer curves of hybrid perovskite–MoS_2_ and pristine MoS_2_ PDs under light illumination and at dark. e–g) Reproduced with permission.^74^ Copyright 2016, Wiley‐VCH. h) Proposed working mechanism of the perovskite/WS_2_ PDs at dark and under illumination. Reproduced with permission.^69^ Copyright 2016, Wiley‐VCH.

As mentioned in Section 3.1.3, 2D materials such as graphene and TMDs have been widely studied and hold great promise for optoelectronic field with unique microstructure and photophysical properties. Cho and co‐workers have first fabricated hybrid perovskite–graphene PDs, and the schematic device architecture is shown in **Figure** **8**a.^70^ A significant PL quenching was observed (see Figure 8b) for the perovskite after the integration of graphene, and this can be explained as follows: The photogenerated excitons in pure perovskite were usually extinguished in picoseconds. However, the presence of graphene enabled an efficient electron transfer to fill the empty states in the valence band of the perovskite, thus resulting in reduced recombination rates and high performances. The transfer curve at a drain voltage of 1 V and a wavelength of 520 nm under different illumination powers is shown in Figure 8c. Upon increasing the incident power, the drain current increased and the Dirac point shifted to positive gate voltage as a result of the negative gating effect caused by the trapped electrons in the perovskite. The resultant *R* value for these PDs was as high as 180 A W^−1^, the EQE reached 5 × 10^4^%, and D* approached 10^9^ Jones. To further elucidate the synergetic effect of perovskite and graphene, a precisely microengineered device was fabricated based on PNWs and monolayer graphene.^71^ During the fabrication process, the ionic OIHP precursor solution not only provided the source materials for crystallization, but also created a highly reducing environment to remove the surface impurities of the graphene. Therefore, the as‐prepared high‐quality PDs achieved an ultrahigh *R* of 2.6 × 10^6^ A W^−1^ at an ultralow illumination power of 3.3 pW, although the rise/decay times were rather high (55 and 75 s, respectively). Apart from providing channels for charge transfer, graphene can also serve as a protecting layer of perovskites against moisture and solvents, as highlighted in Section 3.1.3. Another intensively studied carbon‐based semiconducting material such as CNTs have been also studied incorporated into perovskites.^125^ The corresponding PDs were fabricated by spin coating of a mixed OIHPs precursor solution and single‐walled carbon nanotubes (SWNTs) with different concentrations. The ionic solution enabled a uniform dispersion of the SWNTs and the SWNTs concentration was optimized at 1 wt%. The as‐formed hybrid perovskite/SWNTs thin films demonstrated excellent photoresponse behaviors. As shown in Figure 8d, the transfer curves of the devices showed ambipolar properties under light illumination, and the extracted mobilities of holes and electrons were 595 and 108.7 cm^2^ V^−1^ s^−1^, respectively. These values were much higher than those of pristine perovskite thin films as a result of suppressed charged defects and the reduced scattering effect from trapped charges and the surface roughness. Therefore, these devices afforded superior optoelectronic properties, with *R* of 1.17 × 10^4^ A W^−1^, D* of 3.68 × 10^14^ Jones, *G* of 8 × 10^3^, and rise and decay time of 738 and 912 µs, respectively. Additional carbon‐based semiconducting materials such as reduced graphene oxide (rGO) and C_60_ have been also coupled with OIHPs and their specific performance merits are summarized in **Table** **3**.^126^, ^127^

**Table 3:** Optoelectronic performances of bilayer‐structured perovskites‐based PDs

Device architecture	*R* [A W^−1^]	D* × 10^10^ [Jones]	On/off ratio	*t*_rise_/*t*_decay_ [ms]	EQE [%]	Ref.
Gr./MAPbI_3_	180	0.1	–	87/540	5 × 10^4^	70
Gr./PNWs	2.6 × 10^6^	–	–	5/75	–	71
Gr./MAPbI_3_	115	3 × 10^2^	–	–	–	154
Gr./MAPbBr_2_I	6.0 × 10^5^	–	–	120/750	–	85
MAPbI_3_/rGO	0.0739	–	168	53.5/69.6	–	126
Au NPs/Gr./MAPbI_3_	2.1 × 10^3^	–	–	1800	–	155
PNWs/CNTs	7.7 × 10^5^	–	–	–	1.5 × 10^8^	89
Perovskite/SWNTs	1.0 × 10^4^	3.7 × 10^4^	–	0.738/0.912	–	125
MAPbI_3_/C_60_	–	2.7 × 10^3^	–	0.001/0.0008	80	127
MAPbI_3_/MoS_2_/APTES	2.1 × 10^4^	1.38	–	6170/4500	–	74
MoS_2_/MAPbI_3_	142	26		25/50	3.5 × 10^4^	86
WS_2_/MAPbI_3_	17	10^2^	10^5^	2.7/7.5	–	69
WSe_2_/MAPbI_3_	110	22	–	143/225	2.5 × 10^4^	73
Gr./ MAPbI_3_/Gr.	950	–	500	22/37	–	72
TiO_2_/MAPbI_3_	4.9 × 10^−7^	–	–	20/20	–	156
MAPbI_3_/PW_12_	1.175	–	–	–	–	157
MAPbI_3_/PDPP3T	0.154	8.8	–	–	–	87
MAPbI_3_/RhB	0.0436	–	286	60/40	–	88
MAPbI_3_/C8BTBT	33	–	10^3^	110/800	–	137
MAPbI_3−_ *_x_*Cl*_x_*/P3HT/Gr.	4.3 × 10^9^	–	–	<1000	–	75

Another emerging 2D material TMDs (e.g., molybdenum disulfide (MoS_2_), molybdenum diselenide (MoSe_2_), tungsten disulfide (WS_2_), and WSe_2_) exhibit engaging optical and transport properties such as high light absorption characteristics, long carrier diffusion lengths and high EQE values.^69^ However, atomically thin TMDs hardly absorb light to a sufficient extent, and it is therefore necessary to incorporate them with other materials such as OIHPs to further enhance their performances.^74^ Park et al. have firstly prepared perovskite/MoS_2_ PDs and the corresponding device architecture is shown in Figure 8e.^74^ A MoS_2_ monolayer was obtained by mechanical exfoliation and a perovskite thin film was subsequently formed by spin coating. An analogous PL quenching phenomenon such that represented in Figure 8b took place with a similar charge transfer mechanism (i.e., photogenerated electron–hole pairs in the perovskite were dissociated into free charge carriers at the interface, diffused into the MoS_2_ layer, and collected by MoS_2_–Ti junction (see Figure 8f)). The corresponding devices exhibited an enhanced *I*_light_ eight times larger at *V*_G_ = 20 V compared with pristine MoS_2_‐based devices (see Figure 8g) and showed a high photosensitivity, with a *R* of 4.9 × 10^3^ A W^−1^ and a D* of 8.76 × 10^9^ Jones under a 520 nm wavelength and a 6 mW cm^−2^ incident power. By introducing APTES to induce n‐type doping in MoS_2_, the optoelectronic properties were improved (*R* of 2.11 × 10^4^ A W^−1^ and D* of 1.38 × 10^10^ Jones) under the same testing conditions. It is worth noting that the response time (4.5 s) was significantly shorter than that of its graphene counterpart (75 s). The moisture stability of the devices was further improved via coating with hydrophobic PMMA or octadecyltrichlorosilane/PMMA. Later, the effect of the different MoS_2_ phases (i.e., 1T and 2H) on the electrical performances of hybrid PDs was compared, and results indicated that the metallic 1T phase exhibited exceptionally high *R* (3.1 × 10^3^ A W^−1^) and EQE (7.7 × 10^5^%) values. However, these devices showed low on/off ratios (< 2) and slow response times (0.75 s) due to its metallic conducting nature similar to that of graphene. The semiconducting 2H phase demonstrated reasonable *R* (142 A W^−1^), EQE (3.5 × 10^4^%), and high on/off ratio (≈300) values and fast response time (< 25 ms), rendering it practical for real‐life photodetection.^86^ Besides MoS_2_, heterostructured WS_2_/MAPbI_3_ PDs were recently studied by Wu and colleagues, and they suggested that the perovskite grown on top of atomically flat WS_2_ showed better crystallinity, thereby enhancing the resulting photoresponse to a large extent.^69^ Compared with pristine OIHPs‐based devices, the *I*_light_ of hybrid PDs was enhanced by more than one order of magnitude while the *I*_dark_ was greatly suppressed, leading to ultrahigh an on/off ratio of 10^5^ and a *R* value ≈ 17 A W^−1^. The underlying working mechanism is shown in Figure 8h. The Schottky barriers at the Au/WS_2_ interface and the depletion regions of WS_2_/perovskite interface greatly suppressed the current at dark conditions, while the enhanced charge transfer at the perovskite/WS_2_ interface decreased the Schottky barriers and increased (lowered) the Fermi levels of WS_2_ (perovskite) under light illumination, resulting in high photosensitivity. More recently, novel hybrid WSe_2_/perovskite PDs showed high performances via WSe_2_ laser healing and perovskite modification, exhibiting *R*, EQE, D* values of 110 A W^−1^, 2.5 × 10^4^%, and 2.2 × 10^11^ Jones, respectively.^73^

From the above analysis, it is not difficult to understand the different roles graphene and TMDs have played in the hybrid devices. The graphene acted as charge transport layer to effectively reduce the electron–hole recombination in OIHPs, presenting predominant photogating effect. However, the zero‐bandgap characteristics of graphene usually led to high *I*_dark_, low *I*_light_ /*I*_dark_ ratio, D*, and NEP of the hybrid devices.^55^ The semiconducting TMDs with high charge carrier mobility, tunable optical bandgap, and low driving currents may suppress *I*_dark_ and enable an efficient interfacial charge carrier separation.^69^ The suppressed *I*_dark_ derived from the band alignment between TMDs and perovskite. Specifically speaking, the formation of depletion region at the TMD/perovskite interface as well as the Schottky barrier at the electrode/TMD interface have greatly suppressed the *I*_dark_, and this kind of hybrid devices are more promising in terms of enhanced *I*_light_/*I*_dark_ ratio and response speed.

Apart from 2D materials, 1D materials like ZnO NWs, have also been studied in recent years by integrating with semiconducting materials to realize high‐performance UV detection.^128^, ^129^, ^130^, ^131^, ^132^, ^133^ Wang et al. have fabricated photovoltaic‐like self‐powered ZnO/MAPbI_3_ heterostructured PDs, and systematically investigated the operating mechanism behind them.^128^ A “pyro‐phototronic effect” was proposed in their studies. Three factors including temperatures, UV light intensities, and bias voltages have been discussed thoroughly, and the results indicated that pyro‐phototronic effect was improved at low temperatures, strong UV intensities, and low bias voltages. The output current was greatly enhanced by 500% when the temperature was decreased to 77 K. In addition to the diode‐structured PDs, planar‐structured PDs based on 1D ZnO and perovskites have also been developed recently. Li and co‐workers fabricated for the first time a hybrid planar PDs consisting of electrospun ZnO nanofibers and perovskites.^130^ The devices exhibited a wide range of photoresponse ranging from UV to visible spectrum with an enhanced on/off ratio of 2.8 × 10^3^, a D* of 10^13^ Jones, a response speed of 0.2 s, and an *R* of 0.1 A W^−1^. Herein, the improved overall performance of the hybrid devices was ascribed to the suitable band alignment between ZnO NWs and perovskite, the 1D direct electron transport pathway and a small number of cross‐junctions of ZnO NWs. Zhai and co‐workers have also fabricated ZnO NWs/single crystalline perovskite‐based PDs.^134^ In particular, the cathodoluminescence spectrum was conducted to deeply analyze the surface trap states of the hybrid material, providing guidance for further optimizing the optoelectronic performance. As a result, a decent performance can be obtained, with an *R* of 4.00 A W^−1^, an EQE of 1.3 × 10^3^%, and an excellent flexibility endurance after 200 cycles of bending.

#### 3.2.2 Perovskite/Organic Semiconductor Bilayer Devices

The unsatisfactory performances of OIHPs‐based PDs under the ambient conditions partially result from the instability of OIHPs toward oxygen, moisture and irradiation. Effective and solution‐processable passivation based on the utilization of PMMA, polydimethylsiloxane, or water‐resistant fluoric polymer (CYTOP) has been studied to improve the stability of these devices.^135^, ^136^ The major role of these polymers is to prevent perovskite from external water vapor invasion and oxygen degradation, without altering its intrinsic optoelectronic performances.

**Figure 9:**
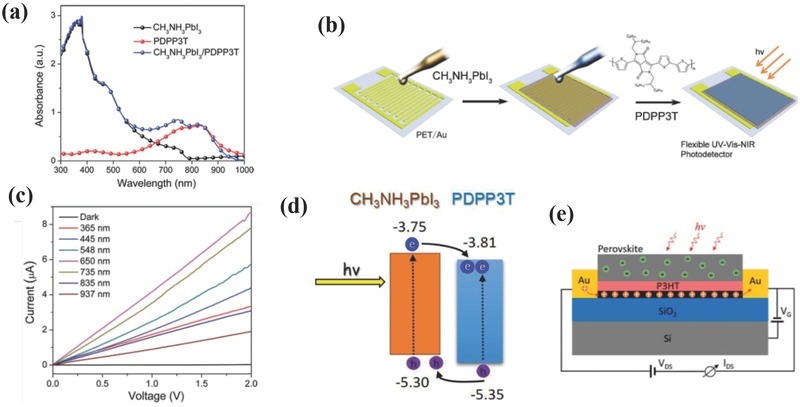
a) UV–vis absorption spectra of MAPbI_3_, PDPP3T, and MAPbI_3_/PDPP3T hybrid thin films. b) Schematic illustration of the fabrication of flexible perovskite/PDPP3T hybrid PDs. c) *I–V* characteristics of the hybrid PDs measured at different wavelengths. d) Energy levels diagram of MAPbI_3_/PDPP3T hybrid PDs. a–d) Reproduced with permission.^87^ Copyright 2016, Wiley‐VCH. e) Schematic diagram of perovskite/P3HT/graphene PDs. Reproduced with permission.^75^ Copyright 2017, ACS.

To further broad the light absorption spectrum of the perovskite, a narrow‐bandgap conjugated polymer (i.e., poly(diketopyrrolopyrrole‐terthiophene), PDPP3T) has been selected as a photosensitizer to extend the UV–vis absorption region of the perovskite to the NIR region, as illustrated in **Figure** **9**a.^87^ Figure 9b shows a simple lateral configuration of the device, which could be achieved by successive spin coating of CH_3_NH_3_PbI_3_ and PDPP3T on prepatterned PET substrates. The as‐prepared hybrid PDs demonstrated a greater photoresponse as compared to the pristine perovskite or PDPP3T devices in terms of low *I*_dark_ (16.4 nA cm^−2^ at 1 V) and enhanced *I*_light_ under different wavelength illumination from 365 to 937 nm (see Figure 9c). The calculated *R* values at 365 (UV), 650 (visible), and 937 nm (NIR) were 10.7, 25.5, and 5.5 mA W^−1^ at 1 V, respectively. These values were one order of magnitude higher than that of the pristine perovskite‐based PDs. The highest D* value reached 1.5 × 10^10^ Jones at 650 nm and 1 V. The fundamental mechanism was described in Figure 9d. Upon illumination by UV–vis light, the excitons were generated within perovskite layer, separated by the bias voltage and collected by the electrodes. With the presence of a perovskite/PDPP3T interface, the excitons were dissociated into free charge carriers more efficiently via band alignment, leading to enhanced photocurrent while suppressing charge recombination and prolonging lifetime of holes. Besides, the hybrid PDs showed high photosensitivity to weak light at different wavelengths, reaching a maximum *R* of 154 mA W^−1^, a D* of 8.8 × 10^10^ Jones, a response time of 50 ms, and an NEP of 21.7 pW Hz^−1/2^. It should not be neglected that PDPP3T not only improved the photoresponse of the perovskite, but also protected the perovskite layer from water and oxygen. Under 25–30% relative humidity in air for 7 d, the hybrid PDs decreased its *R* value by 37% at 650 nm, while the R value of the perovskite‐based PDs dropped drastically at the same conditions, demonstrating an enhanced stability when protected by the PDPP3T layer. Later, the same group have incorporated Rhodamine B (RhB) into perovskite and fabricated them into PDs, and a similar mechanism was put forward to explain the photoresponse enhancement.^88^ The devices achieved an *R* of 43.6 mA W^−1^ at 550 nm, with rise and decay times of 60 and 40 ms, respectively, and an *I*_light_/*I*_dark_ ratio of 286, which were almost comparable to those reported by the above‐mentioned study. Jiang and co‐workers provided another example by utilizing dioctylbenzothieno[2,3‐b]benzothiophene (C8BTBT) as an organic active layer to improve the photosensitivity of perovskite via a gating effect.^137^ In their work, thermal deposition was used to form the C8BTBT layer and the perovskite film, and the resultant PDs showed excellent optoelectronic properties with an *R* as high as 33 A W^−1^, a response time of 100 ms, a mobility of 5 cm^2^ V^−1^s^−1^, and an on‐off ratio at 10^5^, exceeding those of majority of FET‐like PDs. The work mechanism resembled that of previous heterostructured devices, with C8BTBT providing high LUMO (1.7 eV) that blocked the electrons injection more effectively and further reduced the recombination rate of the charge carriers.

By combing inorganic graphene and organic poly(3‐hexylthiophene) (P3HT), a multi‐heterostructured phototransistors was recently fabricated by Yan and co‐workers (see Figure 9e), in which the perovskite acted as a light‐absorbing layer to generate electron–hole pairs, P3HT served as a HTL to dissociate the charge carriers and graphene functioned as a fast channel for charge transfer.^75^ Since the holes were transferred into graphene through P3HT, the accumulated electrons in the perovskite led to a negative gate voltage and induced positive carriers in graphene (i.e., photogating effect). When an ultralow illumination intensity of 14.15 nW cm^−2^ was applied, the measured optoelectronic performance was quite promising. Ultrahigh calculated *R* and *G* values of 1.1 × 10^9^ A W^−1^ and 10^10^ were achieved, which are several orders of magnitude higher than those of previously reported OIHPs‐based PDs. The output performances of perovskite/inorganic and perovskite/organic semiconductor bilayer devices are summarized in Table 3.

### 3.3 Flexible OIHPs‐based PDs

Flexible electronics are of great importance to human‐friendly applications such as stretchable displays, electronic skins, wearable health monitoring devices, and smart textures.^117^, ^138^ Compared with PDs fabricated on rigid substrates such as SiO_2_/Si, flexible PDs have caught the eye of researchers owing to their lightweight and deformable characteristics, and several works have been carried out to explore the relation between the external mechanical forces and the internal optoelectronic performances.^139^, ^140^

**Figure 10:**
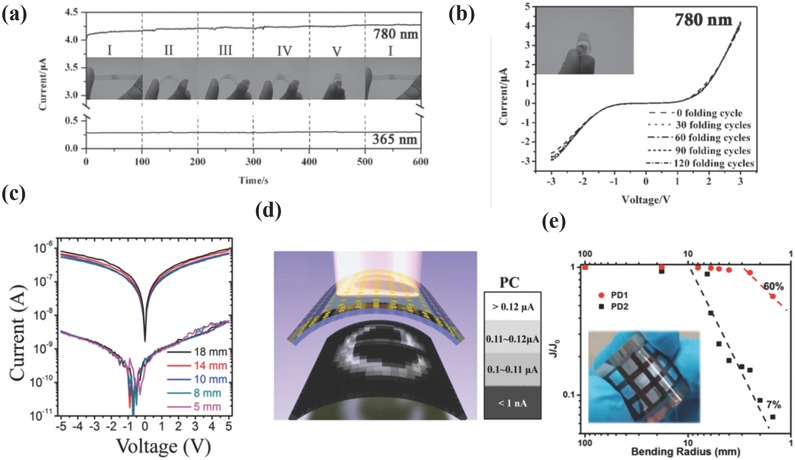
a) *I–t* curves of flexible PDs bended at five different curvatures at 3 V and irradiated by 365 and 780 nm wavelengths. b) *I–V* characteristics of flexible PDs bended at fixed curvatures and different folding cycles. a,b) Reproduced with permission.^80^ Copyright 2014, Wiley‐VCH. c) *I–V* curves of flexible PDs measured in the dark and under 650 nm wavelength illumination at different radii. d) Intensity profile measured by mapping the photocurrent of each pixel at compressive state (*r* = 18 mm). The photocurrents were transformed into four different grey levels. c,d) Reproduced with permission.^142^ Copyright 2016, Wiley‐VCH. e) Photocurrent attenuation of flexible OIHPs‐based PDs based on PEN/Au NWs (PD1) and ITO/PET (PD2) electrodes at zero bias under different bending curvatures. Reproduced with permission.^78^ Copyright 2016, ACS.

As highlighted in Section 3.1.1, Xie and co‐workers demonstrated broad‐band OIHPs‐based PDs fabricated on flexible pre‐patterned ITO/PET substrates, and the devices exhibited outstanding optoelectronic performances in terms of *R*, EQE, on/off ratio, and response time.^80^ Subsequently, the authors studied the electrical stability of these devices by bending them at various curvatures, as shown in **Figure** **10**a. Five bending states were considered, with the *I*_light_ of the devices remaining nearly unchanged when irradiated by different wavelength lasers (365 and 780 nm). As revealed by *I*–*V* characteristics of the devices bent for different folding cycles (see Figure 10b), the *I*_light_ after 120 folding cycles was nearly the same as that of showed by unbent devices. These results indicated that flexible OIHPs‐based PDs could present high folding endurance and electrical stability characteristics, thereby pushing forward the development of flexible optoelectronics. Later, Song and co‐workers studied OIHPs networks by optimizing the material growth process and fabricated them into semitransparent PDs arrays, with high uniformity, photosensitivity and flexibility characteristics.^141^ The as‐synthesized devices showed maximum D* and *R* values of 1.02 × 10^12^ Jones and 0.1 A W^−1^, respectively, with a switching ratio of 300, a response time of 0.3 ms and an equivalent *I*_dark_‐derived shot noise of 4.73 × 10^−15^ A Hz^−1/2^. As for the flexibility properties, the devices showed almost unchanged *I*_light_ values when bent at different angles (θ ≤ 60°) for 1000 times, and the response signals dropped only by 10% even after 10 000 bending cycles at fixed angles, indicating the huge capacity as portable electronics. The authors ascribed these superior performances to the novel network structure of OIHPs that could release part of the bending stress and maintain the device integrity without noticeable cracking.

1D nanomaterials have gained significant attention because of their unique mechanical and electrical properties, which have been discussed in Section 3.1.4. Zhu and co‐workers transformed perovskite thin films into PNWs via a “dissolution‐recrystallization” mechanism and fabricated them into PDs, which exhibited reasonable optoelectronic performances.^118^ With the aim to prove the excellent flexibility characteristics of these materials enabled by the PNW arrays, the devices were bent at a fixed radius (*r* = 0.6 cm) for 200 times and the resulting *I*_light_/*I*_dark_ ratio showed no obvious decay, thereby suggesting the enhanced mechanical integrity and extreme flexibility characteristics. Similarly, aligned single‐crystalline perovskite microwires were synthesized by blade coating, which is suitable for large‐area fabrication of high‐performance and low‐cost optoelectronics on flexible substrates.^142^ By precisely controlling the blading coating speed and the crystallization process, high‐quality perovskite microwires, with a width of 2–3 µm and several centimeters in length, have been successfully prepared. The corresponding devices demonstrated a high photosensitivity (defined as (*I*_light_ − *I*_dark_)/*I*_dark_) of ≈1.34 × 10^4^%, a D* of 5.25 × 10^12^ Jones, and an R of 13.57 A W^−1^. Furthermore, the blade coating method enabled flexible PDs constructed on a PET plastic substrate. Figure 10c depicts the *I*–*V* characteristics of these devices after bending at different radii under dark and 650 nm laser illumination conditions. *I*_light_ and *I*_dark_ remained nearly unchanged, indicating a robust bending stability. To further demonstrate the potential for flexible imaging, a 21 × 21 pixels piece of perovskite microwires‐based PDs was integrated on PET and illuminated by white “e” character under bending state, as shown in Figure 10d. The output currents were tested and grayed into four colors for each pixel, and the “e” character was clearly resolved. The response speed reached 80 and 240 µs for the rise and decay times, respectively. In addition to manufacturing OIHPs into PNWs, electrodes can also be fabricated with NW morphology, and these devices exhibited high conductivity, ultrahigh flexibility, and scalable compatibility.^78^ Patterned Au NWs networks have been in situ prepared on a transparent substrate by etching with a high aspect ratio, and the corresponding photovoltaic‐like OIHPs‐based PDs demonstrated an EQE of 60%, an *R* of 0.321 A W^−1^, an LDR of 84 dB and a response time of 4 µs. To evaluate the flexibility of the Au NWs‐based PDs (PD1), the devices were bent at different radii, showing 60% photocurrent retention at a 1.5 mm radius while the PET/ITO‐based devices (PD2) decreased down to 7% of their original current under the same testing conditions (see Figure 10e), indicating the high stability and mechanical endurance of Au NWs, as well as their promising potential for flexible perovskite optoelectronics.

In addition, the emergence of organic semiconductors (including small molecules and π conjugated polymers) in recent decades have also enabled the development of large‐area and fully flexible devices owing to their lightweight, low cost, and solution processability advantages.^143^, ^144^ For example, flexible MAPbI_3_/PDPP3T‐based PDs were fabricated by a two‐step spin coating method, and high photoresponses have been achieved (see Section 3.2.2).^87^ The flexibility of these devices was studied by bending them at different curvatures and for many times. The *R* values declined by 14% or 17% at a curvature of 4 mm, and maintained 90% or 85% of its original value after 1000 bending cycles at 7 mm curvature radius when irradiated by 650 or 835 nm, respectively, suggesting a remarkable flexible endurance. Similar results were obtained when mixing MAPbI_3_ and RhB, which demonstrated high mechanical flexibility and durability (i.e., a high retention of 92.7% for relative *R* values after 1000 bending cycles at a radius of 9 mm).^88^

## 4 Conclusion and Outlook

PDs based on OIHPs have received tremendous attention in recent years owing to their unique crystalline structures and intriguing photophysical properties. Both internal factors (i.e., chemical composition, crystalline structure, material micromorphology and device architecture, and perovskite/electrode interface) and external factors (i.e., testing conditions and environment) have been discussed. Among these factors, the material micromorphology and device architecture are of paramount importance to the final performance of PDs. Thus, we have carefully discussed single‐component perovskite devices in terms of their different micromorphologies namely, 3D thin‐films and single crystalline, 2D nanoplates, 1D NWs, and 0D NCs. The morphological evolution and various device architecture designs (i.e., vertical‐structured photodiodes and planar‐structured photoconductors and phototransistors) did exert a great influence on the optoelectronic behaviors, which proved that the output performances of PDs were largely determined by internal factors. It is worth noting that the testing conditions (e.g., bias voltages, light intensity power, and wavelengths) and the channel width should also be taken into account to more precisely and objectively evaluate the output performance (especially the photoresponsivity) of different PDs.

Pristine OIHPs‐based PDs usually suffer from low charge carrier mobility, slow photoresponse, and unsatisfactory photosensitivity. Thus, bilayer‐structured OIHPs‐based PDs integrated with inorganic semiconducting (e.g., graphene, CNTs, and TMDs) and organic semiconducting (e.g., PDPP3T, C8BTBT, and P3HT) materials have been thoroughly investigated in order to improve the optoelectronic behavior of pristine OIHPs‐based PDs. The underlying mechanism behind these hybrid devices can be explained in terms of photogating effect (i.e., one of the photogenerated charge carriers in perovskites are extracted into another high‐mobility semiconductor, while the remaining charge carriers act as a gate to amplify the current in perovskites and improve their performances). To further get insight into the potential of perovskites, flexible devices based on these low‐cost and solution‐processable materials have been fabricated and studied, demonstrating excellent mechanical stability, remarkable bending endurance, and holding promise for human‐friendly and portable devices applications.

With regard to the further development of OIHPs‐based PDs, two major issues still remained ahead. One is the intrinsic instability of perovskites that originates from their organic cations. This limitation hampers their practical applications in photovoltaics and also photoelectronics, and may be partially solved by encapsulation techniques commonly employed in the electronic industry, by coupling them with other materials or by replacing organic cations with inorganic cations such as cesium.^30^ Among these strategies, replacement with inorganic ions seems to be more promising since it allows to fundamentally tackling this issue, and according to reported literatures, all inorganic perovskites‐based PDs possessed optoelectronic performances nearly comparable with that of hybrid perovskites.^30^ The second issue is the toxicity of lead to the environment and organisms, which is still hard to address because lead is still indispensable to achieve high performance for OIHPs‐based optoelectronics.^17^ Alternative elements such as tin, germanium, or copper have been proposed to replace lead, although the performances of the resulting devices are far from satisfactory, and the investigations on their photophysical mechanism are still in their infancy. We believe that OIHPs hold a quite bright future in the optoelectronic field and could bring encouraging and inspiring achievements by fully understanding the photophysics of these materials and further optimizing the performance.

## Conflict of Interest

The authors declare no conflict of interest.
